# Emotionally focused couple therapy in cancer survivor couples with marital and sexual problems: a replicated single-case experimental design

**DOI:** 10.3389/fpsyg.2023.1123821

**Published:** 2023-05-02

**Authors:** Selma L. van Diest, Brenda L. den Oudsten, Neil K. Aaronson, Audrey Beaulen, Peter Verboon, Berry Aarnoudse, Jacques J. D. M. van Lankveld

**Affiliations:** ^1^Department of Clinical Psychology, Open University of the Netherlands, Heerlen, Netherlands; ^2^Department of Medical and Clinical Psychology, Tilburg University, Tilburg, Netherlands; ^3^Department of Psychosocial Research, University of Amsterdam, Amsterdam, Netherlands; ^4^Faculty of Health, Medicine and Life Sciences, Maastricht University, Maastricht, Netherlands; ^5^Department of Methodology and Statistics, Open University of the Netherlands, Heerlen, Netherlands; ^6^Consultant, Oisterwijk, Netherlands

**Keywords:** couples therapy, emotionally focused couples therapy, efficacy, single-case experimental designs, cancer, intimacy, relationship dynamics

## Abstract

**Objective:**

The current research examined the effect of Emotionally Focused Couples Therapy (EFCT) on perceived intimacy, affect, and dyadic connection in cancer survivor couples with relationship challenges.

**Method:**

In this longitudinal replicated single-case study, positive and negative affect, intimacy, partner responsiveness, and expression of attachment-based emotional needs were reported every 3 days before and during treatment. Thirteen couples, with one partner having survived colorectal cancer or breast cancer, participated for the full duration of the study. Statistical analysis of the data was performed using randomization tests, piecewise regression, and multilevel analyses.

**Results:**

Adherence to the therapeutic protocol was tested and found adequate. Compared with baseline, significant positive effects on affect variables were found during the therapeutic process. Positive affect increased and negative affect decreased. Partner responsiveness, perceived intimacy, and the expression of attachment-based emotional needs improved, but only in the later phase of treatment. Results at the group level were statistically significant, whereas effects at the individual level were not.

**Discussion:**

This study found positive group-level effects of EFCT on affect and dyadic outcome measures in cancer survivors. The positive results warrant further research, including randomized clinical trials, to replicate these effects of EFCT in cancer survivor couples experiencing marital and sexual problems.

## Introduction

Cancer is a severe stressor in people’s lives and can negatively impact the quality of the intimate relationship, including partnered sexuality, and the ability to connect to the important other (Manne et al., 2010; Sadovsky et al., 2010). In the current study, we aimed to investigate the effects of Emotionally Focused Couple Therapy (EFCT) on crucial aspects of the partner relationship of these couples, using a replicated single-case experimental design (R-SCED).

Receiving a cancer diagnosis and going through the process of diagnostic and curative interventions can have major negative effects on the patient’s physical and mental health and quality of life, as well as on the quality of life of the partner (Fortin et al., 2021; Young et al., 2022). The psychological distress experienced by couples with cancer depends significantly on the intensity of the relational intimacy that partners experience (Manne et al., 2010). Intimacy is related to the way partners discuss their cancer-related concerns, but also by the extent to which they avoid talking about these concerns.

A secure and supportive partner relationship can bolster individuals in dealing with daily distress (Lebow et al., 2012). Problems and tension in one’s relationship, however, can undermine the ability to cope with physical and, in particular, with emotional challenges. The emotion regulation capacity of both partners is considered essential in this respect (Rimé, 2009), and interpersonal stress regulation has been found to be superior to intrapersonal regulation (Levy-Gigi and Shamay-Tsoory, 2017). It is therefore important to identify relational and sexual problems in couples after cancer treatment, and to provide support for these problems, as this will enhance their ability to cope with cancer-related distress.

Emotionally Focused Couples Therapy is an evidence-based approach to improve the quality of partner relationships, that was developed after microanalysis of therapeutic alteration processes (Johnson and Talitman, 1997; Johnson and Wittenborn, 2012; Wiebe and Johnson, 2016; Johnson, 2019b). It is based on the attachment theory, which posits that secure emotional attachment to the partner is a basic need in partner relationships (Hazan and Shaver, 1987; Mikulincer and Shaver, 2007; Péloquin et al., 2014; Sutton, 2019). A secure attachment bond reinforces one’s abilities to be confident, to be creative, to explore the world, and to develop skills to cope with life’s challenges (Johnson, 2019b). In EFCT, conflicts in partner relationships are conceptualized as an interruption of the attachment bond, as a failure in emotion regulation, and as a call for the emotional responsiveness of the partner (Vanhee et al., 2018; Johnson, 2019a). EFCT focuses on rebuilding the attachment bond (Byrne et al., 2004). This aim is pursued through a process comprising three stages: (1) assessment and de-escalation of the conflict pattern, (2) encouraging, supporting, and validating secure attachment experiences between the partners, and (3) consolidation of a new, secure attachment base in everyday life (Byrne et al., 2004). The stages together comprise nine steps, respectively;, assessment of issues, identification of negative interaction cycle, promoting access to unacknowledged feelings, redefining problems in terms of underlying feelings, promoting identification with needs and feelings, promoting acceptance of each other’s experiences, facilitating expression of needs, establishing emergence of new solutions, and consolidating new positions. Process studies have identified two crucial active elements of EFCT for restoring the attachment bond (Lebow et al., 2012). One element involves repairing negative emotional experiences in the first stage of therapy. In the later stage the other element is the creation of new interaction patterns between partners, such as expressing one’s attachment-related needs, and responding to the partner’s needs, for the purpose of improving the couple’s emotion regulation potential.

The main goals of EFCT are expanding constricted emotional responses that prime negative interaction patterns, restructuring interactions to enable partners to become more accessible and responsive to each other, and to foster positive cycles of comfort and caring (Johnson and Zuccarini, 2010). Therefore, the most important outcomes of successful therapy are improved emotion regulation, a stronger attachment bond, and a higher level of perceived intimacy. Partner responsiveness and intimacy were found to be strongly interrelated in partner relationships (Laurenceau et al., 1998; Debrot et al., 2012). Improvements in emotion regulation are represented in this study by decreased negative and increased positive affective responses to the perception of stressful events and situations (Johnson and Talitman, 1997). A stronger attachment bond is reflected by increased expression of emotional needs, and by higher levels of the partner’s responsiveness to these needs (Wiebe et al., 2017). Emotional intimacy involves the experience of strong feelings of closeness, connectedness and bonding (Sternberg, 1986).

The efficacy of EFCT in improving the quality of the intimate relationship as well as emotional resilience has been shown in couples in which one partner was diagnosed with depression (Dessaulles et al., 2003; Denton et al., 2012), with post-traumatic stress disorder, and with anxiety problems (Greenman and Johnson, 2012; Johnson, 2019b). With an average effect size of Cohen’s *d* = 1.3, that was reported in several meta-analyses (Byrne et al., 2004; Wiebe and Johnson, 2016; Rathgeber et al., 2019), EFCT is one of the most effective couple interventions found in the pertinent literature.

However, only a small number of studies have been published about the effects of EFCT in cancer populations. A few case studies described outcomes for individual cancer couples (McLean and Hales, 2010; Adamson, 2013; Grayer, 2016). Naaman (2008) reported improved feelings of connectedness and emotional disclosure in a randomized pilot study of 12 couples treated for breast cancer (Naaman, 2008). McLean et al. (2008) found significant improvements in relationship satisfaction for both patients and partners after EFCT in a pilot study with 16 couples facing terminal cancer. In another RCT conducted by the same research group (McLean et al., 2013) in 42 terminal cancer couples, improved marital functioning was found in both patients and their caregivers after the EFCT intervention when compared to standard care. Nicolaisen et al. (2018) reported positive effects on dyadic adjustment in an RCT among 198 breast cancer couples, after receiving an attachment-based intervention.

Although these findings are promising, further investigations of EFCT in various cancer populations are needed that focus on treatment outcomes that are conceptually related to the attachment theory underlying EFCT, including intimacy, partner responsiveness, and the expression of attachment-based emotional needs. The current study focuses on these treatment outcomes of EFCT in couples affected by colorectal or breast cancer in a R-SCED study. “Single case” here refers to investigating the participant or the couple as the unit of investigation. Participants in single-subject experimental research provide their own control data (Smith, 2012). SCED designs can play an important role in the early phase of intervention development and validation, and are suggested as first choice for testing new treatments, or a validated treatment in a new population, because they allow for significance testing of the treatment effects in each individual, while avoiding the ethical burden for participants of the risk of being allocated to a waitlist condition (Smith, 2012). SCED studies also tie in with the increasing emphasis on tailoring psychological treatment to individual needs, as opposed to general models and treatment modalities designed for larger groups of clients (Wright and Woods, 2020). Replication of the single-case study in a larger group of participants within the same research population provides the ability to investigate intervention effects at the group level. R-SCED designs have been used to investigate outcomes of various interventions, including schema therapy for chronic depression (Renner et al., 2016) and internet-delivered treatment for chronic pain (Wurm et al., 2017).

In the current study, we hypothesized a positive effect of EFCT on emotion regulation, yielding higher positive affect and lower negative affect in the first stage of the treatment (“de-escalation”). We also hypothesized that, in the later stage of treatment (“supporting secure attachment bond,” and “consolidation”), EFCT would promote improvement in the quality of the attachment bond of both partners, measured as higher levels of partner responsiveness, of expression of attachment-based emotional needs, and of perceived intimacy with the partner.

## Materials and methods

### Study design

The current longitudinal study with frequent repeated measurements used a R-SCED to investigate the therapeutic effect of EFCT for couples treated for cancer and experiencing relationship problems. Selected endpoints were emotion regulation, quality of the attachment bond, and perceived intimacy.

### Participants

Eligible participants had completed their primary treatment for colorectal or breast cancer between 12 months and 5 years prior and had not experienced recurrence of their cancer. Additionally, they were between 18 and 75 years of age and had a romantic partner for at least 3 months. Both partners had to agree to undergo treatment. Eligible couples screened positive for relationship distress, as measured using the Maudsley Marital Questionnaire (MMQ; score >20 on the marital dissatisfaction subscale; Arrindell et al., 1983). They also reported lower than average scores on dyadic coping, as assessed using the Revised Dyadic Adjustment Scale (RDAS; score <48; Busby et al., 1995), see Table 1 for psychometric properties of these measures.

**TABLE 1:** Fidelity checks for treatment adherence.

	N	Min	Max	Mean	SD
EFCT fidelity (%)	11	76.71	100.00	93.9	6.32
Inter-rater reliability (%)	11	68.44	98.14	90.39	8.59

Exclusion criteria were high levels of anxiety or depression reported by either partner to not interfere with treatment focus, measured using the Hospital Anxiety and Depression Scale (HADS scores >11) (Zigmond and Snaith, 1983; Bjelland et al., 2002; Mitchell et al., 2010), lack of proficiency in the Dutch language, no access to Internet to fill out the questionnaires, and concurrent treatment focused on improving relational functioning. Couples who were not eligible for participation were informed about other treatment options.

### Procedure

Cancer survivors in 11 Dutch hospitals and cancer centers involved in colorectal and breast cancer treatment were informed by their treating physician of the possibility to apply for participation in the study. Interested couples received information about the research aims, the treatment protocol, and the requirements for participation. Eligible patients then completed baseline assessment of relationship difficulties, intimacy problems, and unsupportive communication, and were asked to confirm if their partner agreed to undergo EFCT treatment and participate in the study. Ethical approval for this study was obtained from the ethical review board of the Zuyderland Hospital in Heerlen, Netherlands.

### Intervention

For the current study, a protocol was developed based on the three stages of EFCT (cycle de-escalation; supporting secure attachment bond; consolidation) and nine steps (Johnson, 2019b). The treatment consisted of 12 sessions taking place within a 20-week period. Affiliated healthcare providers participating in the study were all certified EFCT therapists. The treatment protocol was described comprehensively to maximize homogeneity of the therapeutic intervention across therapists. The therapist reported to the research team when the treatment entered the second stage of therapy (“supporting secure attachment bond”) to mark this moment in the dataset for analysis.

### Assessment

During the 20 weeks of the couple’s participation in the study, both partners were prompted every 3 days by email to complete a 36-item online questionnaire, further referred to as “Affect and Dynamics Questionnaire (ADQ).” Both partners received a prompt on their mobile phones. They were instructed to complete the questionnaire independently and to discuss their responses only after completion, if they desired to do so. The ADQ is comprised of selected items from validated questionnaires, including the Positive and Negative Affect Schedule (PANAS; Crawford and Henry, 2004) and the Personal Assessment of Intimacy in Relationships (PAIR; Schaefer and Olson, 1981), using seven-point Likert scales. The responses ranged from 1 (“not”) through 4 (“moderately”) to 7 (“very”). The intermediate positions had no label. The ADQ measures *positive and negative affect* (“I feel … cheerful/anxious”) (respectively, with 3 and 6 items), *intimacy* with four items (“toward my partner I feel … emotional intimacy”), *desire for sexual intimacy* with two items (“At this moment I feel… sexual desire”), *attachment-based emotional needs* with three items (“At this moment…. I feel the need for my partner’s presence”), and *partner responsiveness* with four items (“At this moment… I receive the emotional support of my partner that I need”). This questionnaire was used in previous research of the research team (van Lankveld et al., 2018).

The 20-item Maudsley Marital Questionnaire (MMQ) was used to measure relationship dissatisfaction, sexual dissatisfaction, and general life dissatisfaction. It uses 9-point Likert scales (range 0–8). The MMQ is organized in three subscales: marital dissatisfaction, sexual dissatisfaction, and general life dissatisfaction. Higher scores indicate greater dissatisfaction. The reference time frame is past 2 weeks. Reliability statistics are satisfactory, with Cronbach’s alpha ranging from 0.60 to 0.88 (Hendriks et al., 1991).

The 14-item Revised Dyadic Adjustment Scale (RDAS) was used to measure quality of relationship functioning. It uses 5- and 6-point Likert scales (range 0–5/0–6). The RDAS is organized in three subscales: consensus, satisfaction, and cohesion. Higher scores indicate greater stability and satisfaction. The reference time frame is “in the current relationship.” Reliability statistics are excellent, with Cronbach’s alpha of 0.90 (Busby et al., 1995).

The 14-item Hospital Anxiety and Depression Scale (HADS) was used to measure levels of anxiety and depression complaints. It uses 4-point Likert scales (range 0–3). The HADS is organized in two subscales: depression, and anxiety. Higher scores indicate greater distress. The reference time frame is past weeks. Reliability statistics are satisfactory, with Cronbach’s alpha ranging from 0.67 to 0.93 (Spinhoven et al., 1997).

**FIGURE 1:**
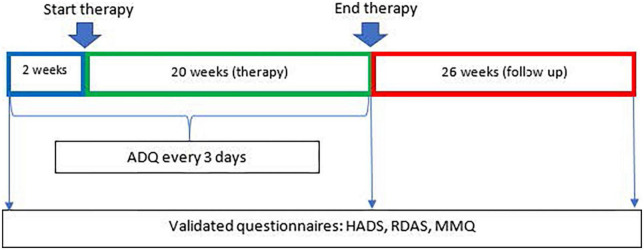
Timeline of measurement moments and start and endpoints of therapeutic intervention.

The questionnaires used to determine eligibility for the study (MMQ, RDAS, HADS) were repeated after treatment ending and after 26 weeks follow up. These questionnaires were used for descriptive purposes only. See Figure 1 for a timeline of the assessments during the research period.

### Treatment fidelity

To examine the level of adherence to the treatment protocol, two master-level students at the Open University, Netherlands, conducted procedural fidelity checks. Audio recordings of treatment sessions were compared to the steps described in the protocol of that particular session, as suggested by Johnson and Wittenborn (2012) using the Emotionally Focused Therapy-Therapist Fidelity Scale [EFT-TFS: Denton et al. (2009). Sessions were selected randomly, audio recorded (after prior consent of the participants)], fully transcribed, and rated according to the EFCT protocol. The students were supervised by a Ph.D. candidate (AB). Eleven sessions were thus recorded and checked. The threshold for sufficient inter-rater reliability (IRR) was set at 80% (Lahey et al., 1983). Ten sessions met this criterion, with IRRs varying between 82 and 98%, with only one session scoring lower (IRR = 68%). All of the rated sessions met the minimum requirement of 75% consistency with the EFCT protocol (proposed by Denton et al., 2009; range 76.7–100%), see Table 1 for fidelity check results.

### Statistical analysis

Confirmatory factor analysis of ADQ data (affect, intimacy, and dyadic coping) was performed to investigate the dimensions of the questionnaire and to confirm the factor structure in the multilevel data set. Correlations between the identified factors were calculated for all outcome measures (positive affect, negative affect, perceived intimacy, expression of emotional needs, partner responsiveness) to understand the underlying associations between the various outcome measures. For these analyses, we used the Lavaan package in R (Schmitt, 2011).

Consistent with the R-SCED design, individual results for each outcome variable were examined to evaluate the therapeutic effects of EFCT. Initially, piecewise regression (PWR) analyses were performed on individual data to evaluate and compare “before” and “during” treatment segments for the five outcome measures (positive affect, negative affect, perceived intimacy, expression of emotional needs, partner responsiveness).

Secondly, data of the couple were compared to assess whether changes on the determined outcome variables in one person would correlate to similar changes for their partner at the same time, using covariance calculations.

Thirdly, for group-level analysis, multilevel PWR (Pustejovsky et al., 2014; Moeyaert et al., 2018) and multilevel randomization tests (Heyvaert and Onghena, 2014; Solmi and Onghena, 2014) were performed on combined data of all participants. Analyses were first performed separately for each outcome variable. Then, using multivariate multilevel tests (randomization test and shift-model analysis), the outcome variables were clustered into a composite *Affect* variable (combining positive affect and mirrored negative affect) and a *Relationship Dynamic* variable (including intimacy, partner responsiveness, and attachment based emotional needs), according to the theoretical EFCT model. The focus in the first stage of EFCT is on de-escalation of negative emotions, and the focus in the subsequent stage is on creating more security in communication, improving the expression of emotional needs, and increasing partner responsiveness. Based on this therapeutic framework, a positive shift in affect level was expected to occur early in the therapeutic process, shortly after start of treatment; the positive shift in the level of the relationship dynamic outcome variable was expected to occur in the second stage of treatment. Hence, the “moment of change” for the statistical analysis for *Affect* was at the start of treatment (immediately following the baseline period), and for *Relationship Dynamic* outcome variables at the start of the second stage of treatment (after baseline and first treatment stage). Significance level for all statistical analyses was 05, unless stated otherwise.

For inclusion in the statistical analysis, participants had to have completed a minimum number of five measurements during baseline and five measurements after the start of treatment (Peng and Chen, 2018; De et al., 2020).

## Results

### Participants

Thirty heterosexual couples were screened for participation. Three couples did not start therapy due to personal circumstances (i.e., too busy with work, travel plans). The average age of the remaining 27 couples was 58.7 years (SD = 10.6; range 37–80 years). The average duration of the relationship was 27.4 years (SD = 15.2; range 4–56 years).

Of the 27 couples that were included for therapy, 21 couples completed all 12 therapy sessions according to the research protocol. The six couples who dropped out attended between one and six sessions. Reasons given for dropout were: end of the romantic relationship, other mental health issues, experiencing the therapy as too intense, or being unable to combine therapy and research requirements with work. Low compliance with assessments, despite repeated prompts to complete the ADQ, resulted in missing data and exclusion of the data of 8 couples from the statistical analyses. Thirteen couples completed the minimum number of five completed measurements during baseline and five measurements after the start of treatment (Peng and Chen, 2018; De et al., 2020).

The average age of these 26 participants was 58.4 years (SD = 12.0) and average duration of the relationship was 28.3 years (SD = 18.5). Nine cancer survivors had colon cancer (seven male and two female), two male survivors had rectal cancer, and two female survivors had breast cancer. The reported pre-treatment anxiety and depression levels were “borderline high” [Anxiety: *M* = 9.5, SD = 4.1; Depression: *M* = 10.4, SD = 3.6; Bjelland et al. (2002)]. Before treatment, participants reported high scores on the marital dissatisfaction subscale of the MMQ [*M* = 22.1; SD = 12.5; Arrindell et al. (1983)], and average scores on the dyadic coping subscale of the RDAS [*M* = 51.4; SD = 6.3; Anderson et al. (2014)], see Table 2. Scores after treatment and 26 weeks follow-up are also reported in this table.

**TABLE 2:** Baseline, end of therapy and follow-up measurement of MMQ, RDAS, HADS.

	Baseline (*N* = 26)		End of therapy (*N* = 15)		Follow-up (*N* = 22)	
	**Mean**	***SD***	**Mean**	***SD***	**Mean**	***SD***
MMQ marital dissatisfaction	22.1	12.5	19.9	8.4	20.2	11.0
MMQ sexual dissatisfaction	23.2	8.1	24.7	9.7	22.3	9.5
RDAS dyadic coping	51.4	6.3	46.6	4.4	46.0	6.0
HADS anxiety	9.5	4.1	8.3	4.3	7.5	2.7
HADS depression	10.4	3.6	10.0	2.9	9.7	3.2

### Factor analysis of ADQ scores

The adequacy of the operationalization of the constructs of positive and negative affect, perceived intimacy, expression of emotional needs, and partner responsiveness was evaluated using Confirmatory Factor Analysis (CFA). The CFA yielded a good model fit for the Affect and Dynamics Questionnaire (ADQ) used in this research. The Chi^2^ (0.000), CFI (0.924), TLI (0.910), RMSEA (0.048), and SRMR (0.051) all exceeded the required thresholds for a good model fit, see Table 3. Correlations within and between the variables are shown in Table 4. Reliability scores within and between variables were found satisfactory, see Table 5.

**TABLE 3:** Factor analysis of ADQ questionnaire.

	*P*	CFI	SRMR	RMSEA
Affect	<0.001	0.954	0.0490	0.106
Intimacy	<0.001	0.979	0.0283	0.109
Dyadic interaction	<0.001	0.926	0.0462	0.153

**TABLE 4:** Correlations within and between outcome measures.

		Negative affect	Intimacy	Attachment based emotional needs	Partner responsiveness
Positive affect	Within group	−0.831*	0.706*	0.023	0.510*
	Between groups	−0.447	0.684*	−0.041	0.765*
Negative affect	Within group		−0.645*	0.018	−0.498*
	Between groups		−0.078	0.014	−0.008
Intimacy	Within group			0.230*	0.725*
	Between groups			0.103	0.791*
Attachment based emotional needs	Within group				0.474*
	Between groups				0.159

*Correlation is significant on 0.01 level; Bold: large-size correlation (α > 0.70).

**TABLE 5:** Reliability test results for outcome measures.

Cronbach’s α	Within variables	Between variables
Positive affect	0.83	0.97
Negative affect	0.75	0.80
Intimacy	0.88	0.97
Attachment based emotional needs	0.71	0.89
Partner responsiveness	0.82	0.96

### Effects of EFCT on affect, intimacy, partner responsiveness, and attachment-based emotional needs

**FIGURE 2:**
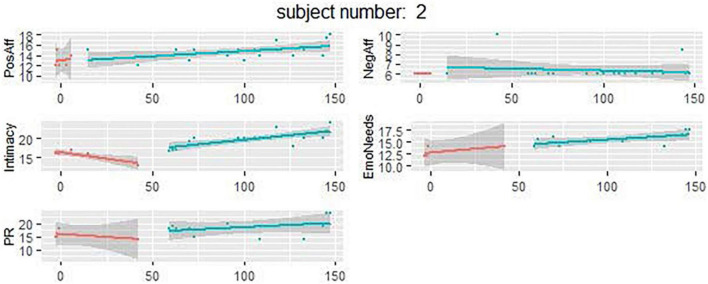
Results of one of the participants showing all outcome measures during the baseline and intervention period. PosAff, positive affect; NegAff, negative affect; EmoNeeds, attachment based emotional needs; PR, partner responsiveness.

As an example, the graphs in Figure 2 show the regression slopes and associated 95% confidence intervals of the endpoint variables during the baseline (first line) and treatment phase (second line) for one randomly chosen participant. See the Supplementary File for data of all participants. Graphs of partners within a couple are displayed next to one another. Many of these graphs indicate upward trends for positive affect, intimacy, partner responsiveness and expression of attachment-based emotional needs and downward trends for negative affect at the individual level. At the individual level, most effects on *positive* and *negative affect*, *intimacy*, *partner responsiveness*, and *expression of attachment-based emotional needs* were not statistically significant, based on the randomization test (see Supplementary Table A). Individual Piecewise Regression (PWR) analyses showed similar non-significant results.

**FIGURE 3:**
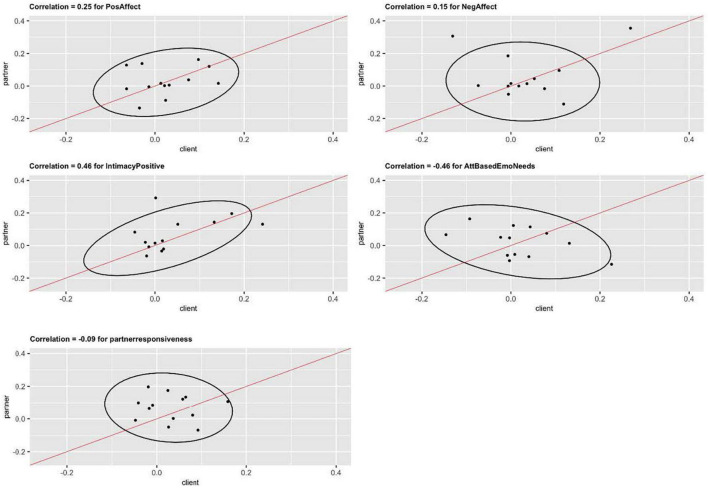
Dyad-level interdependence of patient and partners: correlation of slope distributions for all outcome measures, 95% confidence ellipses for bivariate normal distributions.

To examine effects at the partner level, results within the dyads were compared. Covariances for normal distributions between partners showed medium to large correlations for *positive affect* (*r* = 0.25) and *perceived intimacy* (*r* = 0.46). This indicates that increases in reported *positive affect* in the patient over treatment time tend to be paired with similar increases in positive affect in the partner. Smaller correlations between patient and partner were observed for *negative affect* (*r* = 0.15) and *partner responsiveness* (−0.09). *Attachment-based emotional needs* had a large negative correlation (*r* = −0.46); indicating that increases in expression of attachment-based emotional needs in the one partner tended to be accompanied by a decrease in emotional needs expression in the other partner, see Figure 3 and Table 6.

**TABLE 6:** Dyad covariance results.

Outcome variable	Correlation
Positive affect	0.25
Negative affect	0.15
Intimacy	0.46
Attachment based emotional needs	−0.46
Partner responsiveness	−0.09

At the group level, randomization tests revealed significant treatment effects on *partner responsiveness* and *attachment-based emotional needs* (*p* < 0.014 and *p* < 0.001, respectively). This indicates an improvement on these variables over time from the combined baseline and first phase of treatment to the later phase of treatment. Effects on *positive affect* (*p* = 0.444), *negative affect* (*p* = 0.74), and on *perceived intimacy* (*p* = 0.174) were not significant, see Table 7. PWR analysis was performed for all outcome variables at the group level to test whether the change in slope between the baseline phase and treatment phase was significant. A significant change in favorable direction was found for all outcome variables when comparing the baseline phase with the treatment phase, see Table 7.

**TABLE 7:** Combined randomization and multilevel test outcomes *N* = 26.

	MLA PWR^∧^			Randomization test	
	**Estimate**	**Confidence interval**	***p* <**	**Beta**	***p* =**
Positive affect	0.58	0.14 to 1.02	0.0001*	0.15	0.444
Negative affect	−1.03	−0.43 to −1.64	0.0001*	0.23	0.74
Intimacy	1.20	0.84 to 1.56	0.0001*	0.756	0.174
Attachment-based emotional needs	0.42	0.02 to –0.83	0.0001*	0.237*	0.014*
Partner responsiveness	1.56	1.13 to 1.99	0.0001*	0.442*	0.001*

MLA PWR = multilevel piecewise regression analysis.

*Significant effect *p* < 0.05.

In multilevel PWR analysis the composite Affect and Relationship Dynamics variables both showed a significant change in the expected direction, i.e., Both Affect and Relationship Dynamics improved during treatment, see Table 8. The multilevel randomization test yielded a significant effect for Relationship Dynamics (*p* = 0.002), but the effect on Affect was not significant (*p* = 0.652), see Table 8.

**TABLE 8:** Multilevel piecewise regression analysis (MLA PWR) and randomization test outcomes for *N* = 26.

	MLA PWR			Randomization test	
	**Estimate**	**Confidence interval**	***p***	**Beta**	***P***
Affect	0.805	0.43–1.18	<0.0001*	0.80	0.652
Relationship dynamics	1.08	0.85–1.30	<0.0001*	1.07	0.002*

*Significant effect *p* < 0.05.

## Discussion

In this study, twelve sessions of EFCT were delivered to thirteen cancer survivor couples suffering from relational problems. Tests on individual data showed no significant improvement on any of the outcome variables. However, group level multivariate testing showed positive outcomes of EFCT on Relationship Dynamic variables, in particular on *expression of attachment-based emotional needs* and *partner responsiveness*. The effects on affect variables were not significant. These results imply that EFCT for cancer survivor couples who experience relational and sexual problems has positive effects on the participants’ expression of attachment-based emotional needs and on the responsiveness of partners to each other’s needs. These findings align with other research findings in the field of EFCT (Lebow et al., 2012; Wiebe and Johnson, 2016; Johnson, 2019b), showing that EFCT improves dyadic coping by improving the secure bond between couples.

As per design of the research, all participants who met the criteria of questionnaire completion were included in the data analyses. In a few cases this resulted in the exact minimum number of pre-treatment data points. This low number of data points could thus have lowered the statistical power of the analyses at the individual level, resulting in non-significant outcomes. Because the statistical power increases with larger numbers of participants, this resulted in significant outcomes at the group level, despite the absence of significant findings at the individual level.

Although significant effects were found on dynamic relational patterns, positive and negative affect and perceived intimacy were not found to improve significantly, based on randomization test outcomes. A speculative explanation for the null findings on the affect variables is that our hypothesis that affect would change in the early stage of the therapeutic process is incorrect. For the randomization tests, this hypothesis required comparing baseline data with the data during the full treatment phase. Affect variables did show effects, however, when PWR analysis was performed, indicating significant changes in the slopes of these variables between baseline and treatment phase, but these changes may not have occurred until later in the therapeutic process. This speculative explanation can be seen in the individual participant graphs (see Supplementary material), showing upward trends in positive affect and downward trends in negative affect for most cases throughout the therapeutic intervention.

Emotionally Focused Couples Therapy’s background theory postulates that partner responsiveness and intimacy are closely related factors. This corresponds with our finding of a high correlation (*r* = 0.791) between these factors. Intimacy showed a positive trend in most participants and a stable flat line for others. A possible explanation for the lack of a significant effect on intimacy is that several couples already experienced a high level of intimacy at baseline, leaving less room for improvement. Additionally, potentially other, cancer-related factors, such as physical discomfort or body image issues after surgery, might have interfered with achieving improvements in intimacy. Testing these hypotheses was outside the scope of this study but would add to the understanding of the dynamics of intimacy in couples after cancer treatment.

The current findings, while they can be considered preliminary, suggest support for the putative active elements and dynamic processes in problematic relationships, contained in the adult attachment theory underlying EFCT. The findings suggest that receiving a modest number of 12 therapy sessions, while strictly adhering to the nine-step EFCT program, impact the affective responses involved in emotion regulation. They also suggest that, in the second stage of the program, significant improvements can be achieved regarding dynamic emotion regulation processes, specifically the expression of attachment-based emotional needs and the responsiveness of the partner to such expressed emotional needs. Strengths of the present study were the observed high fidelity of the EFCT treatment and the administration of the therapy by certified therapists. This provides strong confidence that the therapy protocol was closely adhered to. A limitation of this study was that, due to the lack of a control group, conclusions about the causality of the observed effects by the treatment provided cannot be drawn. Although we consider the R-SCED in a limited sample to be adequate in the current early phase of validation of EFCT in a new population of cancer survivor couples (Smith, 2012), the small sample size constitutes another limitation, as it limits the generalizability of the findings to the larger population of cancer survivor couples suffering from relationship difficulties. The small sample also limited the opportunities to perform subgroup analyses, for instance comparing cancer survivors and their healthy partners, or cancer survivors with different diagnoses.

Given the positive outcomes of this pilot study, future research is warranted using a randomized clinical trial design including a waiting list control group. Additional research could also compare the effects of EFCT in couples with different types of cancer. The rich data gathered in studies using R-SCED allow investigation of changes in relationship dynamics before, during, and after completing EFCT. The present study raised several questions regarding the mechanisms of change of EFCT for cancer survivor couples, including “Is there a difference in male vs. female cancer survivors regarding the effect of EFCT for marital problems?” or “Do individuals with a more secure attachment style benefit more or less from the offered therapeutic interventions?” or “Do couples with higher reported relationship distress levels at baseline show more progress in therapy compared to less stressed couples?” Answering these questions requires the assessment of relevant factors in the therapeutic process, including individual and couple characteristics, compliance, and therapeutic working alliance, which could be addressed in future research.

In conclusion, the current study shows promising results regarding the effects of EFCT on intimacy and dyadic coping in colorectal and breast cancer survivors and their partners. Replication studies in larger samples using a randomized controlled research design are warranted.

## Data availability statement

The datasets presented in this study can be found in online repositories. The names of the repository/repositories and accession number(s) can be found below: https://osf.io/yzcf5/?view_only=7f0b383846d14c01a3503e0d643055ae.

## Ethics statement

The studies involving human participants were reviewed and approved by the Medisch Ethische Toetsingscommissie (METC)–Atrium–Orbis–Zuyd. The patients/participants provided their written informed consent to participate in this study.

## Author contributions

SD, JL, BO, AB, BA, and NA contributed to the conceptualization of the study. AB prepared the data collection. SD and PV performed the data analyses. SD, JL, BO, PV, and NA contributed to the writing and reviewing of the manuscript. All authors contributed to the article and approved the submitted version.
