# Supplementary material for: Emotionally focused couple therapy in cancer survivor couples with marital and sexual problems: a replicated single-case experimental design
Source: Front Psychol. 2023 May 2;14:1123821. doi: 10.3389/fpsyg.2023.1123821 (PMC10187887; doi:10.3389/fpsyg.2023.1123821)
Supplement: Supplementary file 14 [file Table_1.docx]

Table A. *Individual p values for all participants and outcome variables*

|  | Positive Affect | Negative Affect | Intimacy | Partner responsiveness | Attachment- based emotional needs |
| --- | --- | --- | --- | --- | --- |
| 1 | 0.747 | 0.118 | 0.211 | 0.261 | 0.592 |
| 2 | 0.317 | 0.128 | 0.363 | 0.255 | 0.189 |
| 3 | 0.060 | 0.830 | 0.758 | 0.162 | 0.102 |
| 4 | 0.720 | 0.150 | 0.151 | 0.186 | 0.321 |
| 5 | 0.243 | 1.000 | 0.277 | 0.554 | 0.621 |
| 6 | 0.108 | 0.358 | 0.678 | 0.517 | 0.745 |
| 7 | 0.248 | 1.000 | 1.000 | 0.293 | 0.958 |
| 8 | 1.000 | 1.000 | 0.575 | 0.541 | 0.310 |
| 9 | 0.656 | 0.159 | 0.708 | 0.267 | 0.706 |
| 10 | 0.417 | 1.000 | 0.365 | 0.388 | 0.646 |
| 11 | 0.811 | 0.424 | 0.596 | 0.077 | 0.683 |
| 12 | 0.193 | 0.033* | 0.810 | 0.852 | 0.419 |
| 13 | 0.643 | 0.118 | 0.376 | 0.087 | 0.558 |
| 14 | 0.953 | 0.447 | 0.670 | 0.428 | 0.503 |
| 15 | 0.338 | 0.075 | 0.440 | 0.456 | 0.488 |
| 16 | 0.929 | 0.030* | 0.079 | 0.244 | 0.382 |
| 17 | 0.931 | 0.159 | 0.354 | 0.417 | 0.501 |
| 18 | 0.778 | 0.359 | 0.562 | 0.716 | 0.719 |
| 19 | 0.412 | 0.751 | 0.572 | 0.284 | 0.056 |
| 20 | 0.972 | 0.327 | 0.464 | 0.474 | 0.363 |
| 21 | 0.071 | 0.903 | 0.571 | 0.578 | 0.527 |
| 22 | 0.026* | 1.000 | 0.261 | 0.156 | 0.118 |
| 23 | 0.026* | 0.192 | 0.383 | 0.408 | 0.492 |
| 24 | 0.896 | 0.651 | 0.297 | 0.334 | 0.403 |
| 25 | 0.192 | 1.000 | 0.560 | 0.524 | 0.224 |
| 26 | 1.000 | 0.435 | 0.745 | 0.111 | 0.362 |

*Note:* * significant effect p < .05
